# EFFECTS OF A ONE-YEAR PIANO PRACTICE INTERVENTION ON COGNITIVE FLEXIBILITY IN HEALTHY OLDER ADULTS

**DOI:** 10.1093/geroni/igae098.2188

**Published:** 2024-12-31

**Authors:** Melanie Mack, Damien Marie, Florian Worschech, Tillmann Krueger, Christopher Sinke, Eckart Altenmueller, Clara James, Matthias Kliegel

**Affiliations:** University of Geneva, Geneva, Geneve, Switzerland; University of Applied Sciences and Arts Western Switzerland HES-SO, Geneva School of Health Sciences, Geneva Musical Minds lab, Geneva, Geneve, Switzerland; Hanover University of Music, Drama and Media, Hannover, Niedersachsen, Germany; Division of Clinical Psychology & Sexual Medicine, Department of Psychiatry, Social Psychiatry and Psychotherapy, Hannover Medical School, Hannover, Niedersachsen, Germany; Division of Clinical Psychology & Sexual Medicine, Department of Psychiatry, Social Psychiatry and Psychotherapy, Hannover Medical School, Hannover, Niedersachsen, Germany; Hanover University of Music, Drama and Media, Hannover, Niedersachsen, Germany; University of Applied Sciences and Arts Western Switzerland HES-SO, Geneva School of Health Sciences, Geneva Musical Minds lab, Geneva, Geneve, Switzerland; Centre for the Interdisciplinary Study of Gerontology and Vulnerability, University of Geneva, Geneva, Geneve, Switzerland

## Abstract

We analyzed data of a randomized controlled trial to examine the effects of piano practice on cognitive flexibility in healthy older adults. Participants (N = 153, 69.5 ± 3.5 years of age, 57.5% females) were randomly assigned to a piano practice group (PP) or a control group engaged in active music listening (MC). Both groups underwent a year-long intervention with weekly 60-minute lessons and daily homework. We assessed switch and mixing costs in terms of speed (mean RTs) and variability (standard deviation of RTs) with a number switch and a perceptual switch task. We employed MIDI-based Scale Analysis to assess pianistic performance. Tests were conducted at baseline, after 6 months, post-intervention (12 months), and at follow-up (18 months). Results revealed more pronounced improvements in pianistic performance in the PP compared to the MC group over the course of the intervention. Both groups exhibited gains in several cognitive flexibility outcomes, which originated primarily in the latter half of the intervention. For mixing costs, the PP group showed partly greater improvements compared to the MC group. Additionally, the findings indicated a compensation account in both groups, which was partially more pronounced in the PP group. This study suggests that both piano practice and active music listening – with piano practice to a slightly higher degree - enhance cognitive flexibility, particularly in sustained control mechanisms linked to mixing costs. Both interventions potentially require longer than 6 months to induce behavioral transfer effects and are especially beneficial for individuals with lower cognitive flexibility levels.

